# Cutaneous body image in patients with hidradenitis suppurativa: a hospital-based cross-sectional study

**DOI:** 10.1038/s41598-023-35120-9

**Published:** 2023-05-16

**Authors:** Dimitra Koumaki, Evangelia Rovithi, Erato Solia Apokidou, Marios Papadakis, Alexander Katoulis, George Evangelou

**Affiliations:** 1 Dermatology Department, University Hospital of Heraklion, 71110 Heraklion, Crete, Greece; 2 Department of Internal Medicine, Agios Nikolaos General Hospital, Knosou 4, Ag. Nikolaos, 72100 Crete, Greece; 3 Department of Surgery II, Witten/Herdecke University, Heusnerstrasse 40, 42283 Witten, Germany; 4 2nd Department of Dermatology and Venereology, Medical School, National and Kapodistrian University of Athens, “Attikon” General University Hospital, Chaidari, Athens, Greece

**Keywords:** Psychology, Diseases, Medical research

## Abstract

Hidradenitis suppurativa (HS) has been linked with body image (BI) impairment and reduced quality of life (QoL). We sought to evaluate the associations between Cutaneous Body Image Scale (CBIS) and disease severity in HS patients.Between July 2020 and January 2022, a cross-sectional study was carried out including consecutive HS patients above the age of 16 who attended a Tertiary Referral Hospital in Greece. Disease Severity was graded with the Hurley stage, HS-Physician’s Global Assessment (HS-PGA) scale, and the Modified Sartorius scale (MSS). Patients completed at their first visit ten survey instruments including Patients’ Severity of disease, pain and pruritus scale, CBIS, Multidimensional Body-Self Relations Questionnaire (MBSRQ) including 5 subscales: Appearance Evaluation (AE), Appearance Orientation (AO), Body Areas Satisfaction Scale (BASS), Overweight Preoccupation (OWP), and Self Classified Weight (SCW) , Dermatology Quality of Life Index (DLQI), Skindex-16, EQ5D 5L, EQ- visual analogue scale (VAS), PHQ9, and GAD7. In total, 70 HS patients above 16 years old participated, mean [SD] age, 34.44 [11.64] years; 49/70 (70%) males and 21/70 (30%) females. Mean ± SD CBI, DLQI, Skindex-16 total, EQ-5D-5L, EQ VAS, PHQ9 and GAD7 were 5.59 ± 1.58, 11.70 ± 8.88, 52.90 ± 27.75, 0.75 ± 0.21, 62.48 ± 21.12, 7.64 ± 5.56, 7.87 ± 5.23 respectively. Moderate to severe CBI dissatisfaction was reported by 36/70 (51.42%) patients. CBI was correlated with appearance evaluation (AE) (*p* < 0.01, r = 0.544), body areas satisfaction (BASS) (*p* < 0.01, r = 0.481), and overweight preoccupation subscale (OWPS) (*p* < 0.01, r =  − 0.267), and Skindex-16 (*p* < 0.01, r =  − 0.288). HS patients with affected genital areas scored higher in disease patient’s severity score (*p* = 0.015), and male patients scored higher in Skindex-16 than females(*p* < 0.01). Our study found that the mean of CBI in HS patients was 5.59 ± SD 1.58. Predictors for CBI dissatisfaction were low scores of MBSRQ Appearance Evaluation (AE) and Body Areas Satisfaction Subscale (BASS).

## Introduction

Hidradenitis suppurativa (HS) is a chronic inflammatory skin condition that affects the terminal hair follicles mainly of the intertriginous skin areas of the axillary, groin, perianal, perineal, and inframammary regions, and is characterized by the presence of recurrent painful nodules on these areas^1^. HS patients are often negatively perceived and stigmatized^2^, leading to impaired Quality of Life (QoL)^3,4^, poor Body Image (BI)^5,6^, and higher incidence of psychiatric disorders^7^, anxiety, and depression^8,9^. Cutaneous body image (CBI) may be defined as the individuals’ mental perception of the appearance of their integumentary system, i.e., their skin, hair, and nails^10^. Cutaneous body image scale (CBIS) is an important dermatologic construct—both in the wide range of cosmetically disfiguring skin disorders where CBI dissatisfaction can have a profound impact on the quality of life (QoL) of the patient and in situations where the CBI is distorted such as body dysmorphic disorder^10,11^. It is a seven-item scale^10^. Patient dissatisfaction with CBI is often the primary consideration in deciding whether or not to commence treatments for some skin disorders^11^. Because HS causes disfigurement mainly of the intertriginous skin areas of the axillary, groin, perianal, perineal, and inframammary regions, we expected impaired CBI in these patients. This study aims to evaluate CBI in HS patients and whether disease severity, the topography of lesions, age at onset, disease duration, obesity, general health, anxiety, and depression are linked to CBI impairment.

## Materials and methods

This was a prospective, non-interventional, observational cross-sectional study conducted at a Tertiary Referral Hospital in Greece. Consecutive patients with hidradenitis suppurativa (HS) above the age of 16, willing to participate in the study, after obtaining informed consent for subjects above the age of 18 or with parental consent for patients below the age of 18, were recruited from the Dermatology Department at their first visit between July 2020 and January 2022. The inclusion criteria were i) age above 16, ii) willingness to participate together with parental consent for patients below the age of 18, iii) a clinical diagnosis of HS made by an experienced dermatologist, and iv) capability of completing the QoL questionnaires that were administered. Ethics committee approval was obtained for this study and informed written consent was provided from all participants. Procedures followed were by the ethical standards of the responsible institutional ethics committee and with the Helsinki Declaration of 1975, as revised in 1983.

All participants were examined by the same dermatologist who provided information about clinical characteristics, affected body areas, past medical history, comorbidities, and treatments. Disease severity was assessed by the clinician with the following three measures: Hurley staging, Hidradenitis Suppurativa-Physician’s Global Assessment (HS-PGA) of disease, and Modified Sartorius Score (MSS)^12^. Patients were also asked to rate their a) HS disease severity using a scale from 0 to 10, where 0 indicated clear disease and 10 represented very severe disease^13^, b) HS-related pain intensity on a scale from 0 to 10, where 0 indicated no pain at all and 10 pain as bad as it could be^14–16^, and c) HS-related pruritus severity using a scale from 0 to 10, where 0 indicated no pruritus and 10 represented very severe pruritus because of HS lesions^16,17^. All participants completed seven paper-based questionnaires (Table 1): the CBIS, the Multidimensional Body-Self Relations Questionnaire-Appearance Scales (MBSRQ-AS) that contains the five appearance-related items: Appearance Evaluation (AE), Appearance Orientation (AO), Body Areas Satisfaction Scale (BASS), Overweight Preoccupation (OWP), and Self Classified Weight (SCW) items^18–20^, two skin-specific Quality of Life (QoL) instruments: Dermatology Life Quality Index (DLQI)^21^ and Skindex-16^22^, a general General Health-Related Quality of Life, HRQoL, : the EQ-5D-5L and EQ visual analog scale (EQ-VAS)^23–26^, Patient Health Questionnaire-9 (PHQ-9) for assessing depression^27,28^ and GAD-7 (Generalized Anxiety Disorder 7)^29,30^ for screening for anxiety (See supplementary file 1).

**Table 1:** Brief description of the main quality of life questionnaires (QoL) used in our study.

Questionnaire used	What is explored	Number of items	Likert scale answer for each question
CBIS	Cutaneous body image scale	7 questions	0–9
MBSRQ-AS	Body image	34 items	1–5
AE	Appearance evaluation subscale	7 items	1–5
AO	Appearance orientationsubscale	12 items	1–5
BASS	Body areas satisfaction scale	9 items	1–5
OWP	Overweight preoccupation subscale	4 items	1–5
SCW	Self-classified weight subscale	2 items	1–5
DLQI	Dermatology life quality index ( Skin specific quality of life instrument)	10	0–3
Skindex-16	Skin-specific quality of life instrument	16	7 possible answers for each question
PHQ-9	Patient health questionnaire-9 ( measuring depression)	9	4 possible answers for each question
GAD-7	General anxiety disorder-7 ( evaluating anxiety)	7	4 possible answers for each question
EQ5D 5L	EuroQol- 5D-5L ( general health quality of life questionnaire)	5	5 possible answers for each question
EQ VAS	EQ visual analogue scale (general health visual analogue score)	A numerical scale from 0–100	0–100

### Statistical analysis

Baseline patient demographics, disease characteristics, and comorbidities were summarized descriptively with a mean (SD) for continuous variables and counts and percentages for categorical variables. Non-parametric Mann–Whitney *U* and Kruskal–Wallis *H* tests were used to compare patient- and physician-based outcome scores. Analysis of the relationship between continuous variables was accomplished by calculating Spearman’s correlation coefficients (weak correlation: rs < 0.2; moderate correlation 0.4 ≤ rs < 0.6; strong correlation 0.6 ≤ rs ≤ 1). The significance threshold was adjusted with Bonferroni correction statistical test. The association between CBIS and continuous variables was also studied using logistic regression analysis. All statistical tests were performed using SPSS for Windows v25. The threshold statistical significance was set at a = 0.05 for all tests.

### Ethical approval

Approval was obtained from the Ethical Review Board of the University Hospital of Heraklion, Heraklion, Crete. The methods used in this study comply with the principles of the Declaration of Helsinki.

### Consent to participate

All participants gave their explicit written informed consent for participation in this study. For participants with age below 18 years old, also their legal guardians consented to participate in the study.

## Results

A total of 70 consecutive HS patients with active disease, at least one flare over the last 6 months, were recruited from the Outpatient Clinics of the Dermatology Department of a Tertiary Referral Hospital in Greece, between July 2020 and January 2022. There were 21/70 (30%) females and 49/70 (70%) males. The mean age of HS patients was 34.33 ± Standard Deviation (SD) 11.64 years, the mean age of onset of HS was 22.33 ± SD 7.60 years, and the mean duration of disease was 12.11 ± SD 9.53 years. Hurley stage I was 13/70 (18.6%), Hurley stage II 41/70 (58.6%), and Hurley stage III 16/70 (22.9%) of HS patients. Baseline demographic and disease characteristics and comorbidities are shown in Table 2.

**Table 2:** Patients’ demographics, clinical characteristics and quality of life questionnaires in our study's 70 patients with Hidradenitis Suppurativa (HS) and correlations with Cutaneous Body Image Scale (CBIS).

	Mean ± SD (Standard Deviation)	Median	95% C.I. (Confidence Interval)	*p* value	*r* correlation coefficient	Bonferroni correction	Linear regression analysis
Gender	Number, (%)			*P* = 0.34	0.116	*P* = 0.3	
Female N (%)	21/70 (30%)						
Male N (%)	49/70 (70%)						
	Mean (± SD**)**						
Age (years), mean (SD)	34.44 (± 11.64)	34.00	31.67–37.22	*P* = 0.54	− 0.074	*P* = 0.5	
Age of onset of HS, mean (SD)	22.33(± 7.60)	20.00	20.52–24.14	*P* = 0.55	− 0.071		*P* = 0.55
Age of diagnosis of HS, mean (SD)	30.86 (± 10.38)	30.00	28.38–33.33	*P* = 0.21	− 0.152		
Age of onset of treatment, mean (SD)	31.49 (± 9.93)	30.00	29.12–33.85	*P* = 0.15	− 0.172		
Duration of disease in years, mean (SD)	12.11 (± 9.53)	10.50	9.84–14.39	*P* = 0.78	− 0.033		
Patient’s severity of disease, mean (SD)(On a scale from 0 to 10)	6.20 (± 2.59)	7.00	5.58–6.82	*P* = 0.84	− 0.019		
Patients’ pain severity, mean (SD)(On a scale from 0 to 10)	4.80 (± 3.60)	5.50	3.94–5.66	*P* = 0.70	− 0.047		
Patients’ pruritus severity, mean (SD)(On a scale from 0 to 10)	3.29 (± 3.31)	2.50	2.50–4.08	*P* = 0.45	− 0.092		
Disease Severity Scores, mean (SD)							
Sartorius score, mean (SD)	67.06 (± 61.87)	47.00	52.31–81.81	*P* = 0.82	− 0.027		*P* = 0.8
Hurley stage				*P* = 0.34	− 0.116	*P* = 0.3	
Hurley stage I, N (%)	13/70 (18.6%)						
Hurley stage II, N (%)	41/70 (58.6%)						
Hurley stage III, N (%)	16/70 (22.9%)						
HS-PGA score				*P* = 0.91	− 0.014	*P* = 0.9	
Clear, N (%)	1/70 (1.4%)						
Minimal, N (%)	9/70 (12.9%)						
Mild, N (%)	33/70 (47.1%)						
Moderate, N (%)	17/70 (24.3%)						
Severe, N (%)	6/70 (8.6%)						
Very severe, N (%)	4/70 (5.7%)						
Smoking status				*P* = 0.05	− 0.13	*P* = 0.05	
Current smoker, N (%)	43/70 (61.4%)						
No smoker, N (%)	20/70 (28.6%)						
Ex-smoker, N (%)	7/70 (10%)						
	Mean (± SD)	Median	95% C.I				
BMI	30.02	29.96	28.52–31.53	*P* = 0.62	− 0.16	*P* = 0.62	
Normal BMI, N (%)	14/70 (20%)						
Overweight, N (%)	20/70 (28.6%)						
Obese, N (%)	36/70 (51.4%)						
Current treatment				*P* = 0.94	0.14	*P* = 0.9	
No treatment, N (%)	21/70 (30%)						
Topical treatment, N (%)	21/70 (30%)						
Oral treatment with antibiotics, N (%)	22/70 (31.4%)						
Adalimumab treatment, N (%)	6/70 (8.6%)						
Marital status				*P* = 0.73	− 0.042	*P* = 0.7	
Married, N (%)	33/70 (47.1%)						
Single, N (%)	34/70 (48.6%)						
Divorced, N (%)	3/70 (4.3%)						
Employment status				*P* = 0.555	0.072	*P* = 0.5	
Pupil, N (%)	2/70 (2.9%)						
Student, N (%)	13/70 (18.6%)						
Employed, N (%)	41/70 (58.6%)						
Unemployed, N (%)	13/70 (18.6%)						
Retired, N (%)	1/70 (1.4%)						
Educational level				*P* = 0.052	0.062	*P* = 0.05	
Primary school, N (%)	2/70 (2.9%)						
Secondary school, N (%)	29/70 (41.4%)						
Technical studies, N (%)	16/70 (22.9%)						
University level, N (%)	23/70 (32.9%)						
Skin phototypes				0.23	0.144	0.23	
Skin type I, N (%)	0/70 (0%)						
Skin type II, N (%)	4/70 (5.7%)						
Skin type III, N (%)	55/70 (78.6%)						
Skin type IV, N (%)	11/70 (15.7%)						
Cutaneous Body Image Scale (CBIS) total	5.97 ± 1.58	6.14	5.59–6.35				
CBI dissatisfaction							
Severe dissatisfaction with CBI, N (%)	1/70 (1.4%)						
Moderate dissatisfaction with CBI, N (%)	33/70 (47.1%)						
Mild to none dissatisfaction with CBI, N (%)	36/70 (51.4%)						
Body Image Scale							
Appearance evaluation subscale, mean ± SD	3.03 ± 0.79	3.14	2.84–3.22	***P* = 0.00**	**0.544**		*P* < 0.01
Appearance orientation subscale, mean ± SD	3.32 ± 0.52	3.33	3.11–3.36	*P* = 0.34	− 0.116		*P* = 0.3
Body areas satisfaction subscale, mean ± SD	3.19 ± 0.73	3.22	3.02–3.37	***P* = 0.00**	**0.481**		*P* < 0.01
Overweight preoccupation subscale, mean ± SD	2.73 ± 0.79	2.75	2.54–2.92	***P* = 0.02**	− **0.267**		*P* < 0.01
Self-classified weight subscale, mean ± SD	3.76 ± 0.85	4.00	3.55–3.96	*P* = 0.24	− 0.141		*P* = 0.2
DLQI total (0–30), mean ± SD	11.70 ± 8.88	11.00	9.58–13.82	*P* = 0.52	− 0.078		*P* = 0.5
Skindex-16 Total, (0–100) mean ± SD	52.90 ± 27.75	48.43	46.28–59.52	***P* < 0.01**	− **0.288**		*P* < 0.01
Skindex-16 Symptoms, mean ± SD ( 4 items)	49.94 ± 28.45	50.00	43.15–56.72	*P* = 0.31	− 0.121		*P* = 0.3
Skindex-16 Emotions, mean ± SD (7 items)	60.91 ± 30.06	65.00	53.74–68.08	***P* = 0.01**	− **0.302**		*P* < 0.01
Skindex-16 Functioning, mean ± SD (5 items)	44.04 ± 33.34	40.00	36.09–51.99	***P* = 0.01**	− **0.305**		*P* < 0.01
PHQ9, mean ± SD	7.64 ± 5.56	7.00	6.32–8.97	*P* = 0.31	− 0.121		*P* = 0.3
GAD7	7.87 ± 5.23	6.00	6.62–9.12	*P* = 0.98	− 0.002		*P* = 0.9
EQ5D 5L, mean ± SD	0.75 ± 0.21	0.81	0.70–0.80	*P* = 0.81	0.029		*P* = 0.8
EQ VAS, mean ± SD	62.48 ± 21.12	60.00	57.44–67.52	*P* = 0.66	0.053		*P* = 0.6

A *p* value less than 0.05 was considered statistically significant. The correlation coefficient (r value) was considered strong, moderate, and weak for values above 0.6, between 0.2 and 0.6, and below 0.2 respectively.

*HS* Hidradenitis suppurativa, *SD* Standard deviation, *C*.*I*. Confidence interval, *CBIS* Cutaneous body image scale, *DLQI* Dermatology life quality index, *PHQ-9* Patient health questionnaire-9, *GAD-7* General anxiety disorder-7, *EQ5D 5L* EuroQol- 5 Dimension, *EQ* VAS Visual analogue scale, *r* Correlation coefficient, *p* value Probability value, *C*.*I*. Confidence interval, *SD* Standard deviation, *N* Number, *BMI* Body mass index, *PMH* Past medical history, *IBD* Inflammatory bowel syndrome, *HS-PGA* Physicians’ Global Assessment of HS severity.

Significant values are in bold.

The mean value of CBIS in our 70 HS patients was 5.97 ± 1.58 (Table 2). 36/70 (51.4%) had mild dissatisfaction with CBI, 33/70 (47.1%) had moderate dissatisfaction and 1/70 (1.4%) had severe dissatisfaction with their CBI (Table 2). Mean ± SD CBIS, DLQI, Skindex-16 total, EQ-5D-5L, EQ VAS, PHQ9 and GAD7 were 5.59 ± 1.58, 11.70 ± 8.88, 52.90 ± 27.75, 0.75 ± 0.21, 62.48 ± 21.12, 7.64 ± 5.56, 7.87 ± 5.23 respectively.

The fixed effects of each demographic and clinical factor on the CBIS, MBBS, anxiety, depression, EQ5D5L, EQVAS, DLQI, and overall Skindex-16 score within each domain score were investigated using univariate and multivariate analysis.

Multiple linear regression showed that CBI dissatisfaction was correlated with MBSRQ AE (*p* < 0.01), and BASS (*p* < 0.01). BMI was a predictor for AE (*p* < 0.01), BASS (*p* = 0.02), SCW (*p* < 0.01), Skindex-16 total (*p* = 0.02), and depression (*p* = 0.03). HS pain severity was also a predictor of depression (*p* < 0.01).

Furthermore, HS patients with Hurley stage 3 had a lower EQVAS (*p* = 0.01) than the others indicating probably a lower overall assessment of their health. HS male patients scored higher in Skindex-16 than females. HS patients with affected genital areas considered their skin disease more severe as they scored higher in the patient’s disease severity score (*p* = 0.01).

Smokers had a younger age of HS onset (*p* = 0.02) while they scored higher in pain (*p* < 0.01), quality of life as measured by Skindex-16-total (*p* < 0.01), EQVAS (*p* < 0.01), and depression assessed by PHQ-9 (*p* < 0.01).

## Discussion

In this study, we assessed CBIS in HS patients. We also evaluated the relationship between CBIS and demographic and clinical parameters and Health-Related Quality of Life measures among HS patients, revealing important information that may inform our approach to this group of patients. In our study, the mean CBI value was 4.11 ± SD 2.72. A previous study on 127 dermatology patients conducted in Canada reported a mean ± SD CBIS score (possible variation from 0 to 9) of 4.44 ± 1.56. In this sample 28.3% had acne, 21.3% had psoriasis, 40.1% had onychomycosis/athletes’ foot, and 10.3% had atopic dermatitis/alopecia/other. The mean CBIS was much decreased (*P* = 0.004) than the 4.96 ± 1.73 scores in the 312 persons belonging to the community-based nonclinical group, consistent with greater body image dissatisfaction in the dermatology group^10,11^. Likewise, in a study conducted in Japan, the CBIS values in dermatology patients (3.18 ± 1.69) were much lower than those among healthy individuals (4.11 ± 1.80)^31^. Probably, the lower mean scores in Japanese individuals than those in Canadian suggest that the Japanese group had greater CBI concerns. Previous studies showed a correlation between CBI and the body dissatisfaction subscale of the Eating Disorder Inventory^10^, global and appearance-related self-esteem^32^, negative experiences of skin disease (embarrassment and bullying), and age^33^.

In our study, CBI dissatisfaction was correlated with AE and BASS, while patients with genital areas affected scored higher in patient severity of the disease. BMI was a predictor for Skindex-total, AE, BASS, SCW, and depression. Previous studies have shown that HS patients have frequently reduced quality of life^3,4^, depression^8,9^, and impaired Body Image (BI) compared with healthy controls^5,6^.

A psychological condition called alexithymia is characterized by a lack of emotional expression, description, and awareness^34^. There is evidence linking HS to alexithymia, broadening the range of psychological diseases linked to HS^34^. Patients with HS may be affected by the phenomenon of fear of stigmatization and exclusion^35^. patients find their skin lesions to be extremely uncomfortable and unattractive, believe they are unclean, and feel embarrassed about it^35^. They are well aware of how unpleasant the discharge of their nodules and pustules smells. When they are heavily discharged, they don't look for companionship and instead stay alone at home^35^.

According to Schmid-Ott et al., stigmatization experiences vary^36^. Those who have visible lesions endure a greater degree of stigmatization than those who have unseen lesions^36^. Patients with HS fall between the visible and unseen, discredited and discreditable categories^35^. These events may eventually affect patients' self-perceptions, resulting in internalized skin bias (ISB)^37^. Recently, a questionnaire evaluating internalized skin bias (ISB) has been validated in HS patients^37^. Stigmatization may result in impaired body image perception^38^. Patients with HS internalize society's judgments, which could have a severe impact on their ability to access medical care^39^. In order to improve the quality of health care, it is crucial to address internalized stigma in addition to disease activity^39^. A psychodermatological approach enhances QoL, disease flare-ups, and long-term management of the condition^40^.

Limitations of this study should be considered. First, because HS patients recruited from a single center in a tertiary referral hospital may differ from those in practice, therefore the generalizability of the study results may be limited. Second, our study enrolled a limited number of HS patients and there was no control group. Participants were only examined at a single time point. Future studies should collect longitudinal data over multiple points in time as this approach may yield information on how CBI changes or remains stable over time and depending on treatment response.

In conclusion, we recommend the use of CBIS across various settings, including clinical care and clinical research in HS patients. Regular evaluation of the CBI could be beneficial because it validates the patients’ concerns about their CBI as being clinically relevant. The medical practitioner may wish to document whether a significant discrepancy exists between the patient’s subjective evaluation of his or her cosmetic problem and an objective dermatologic evaluation of the cosmetic effect of the skin disorder because this could be an indication of body image pathology or other underlying psychiatric comorbidities. Patient dissatisfaction with CBI is often the primary consideration in deciding whether or not to commence treatment for various skin conditions. Assessment of CBI in the dermatology patient is best performed using a biopsychosocial model that involves assessment of concerns about the appearance of the skin, hair, and nails, assessment of comorbid body.image pathologies, and assessment of other psychiatric comorbidities. Future prospective studies before and after treatment in a large cohort of patients with HS are needed to provide more solid results.

## Conclusions

Our findings might have some practical applications. We believe that employing the use of CBIS in clinical settings, will assist dermatologists to understand HS patients’ expectations and needs. Upcoming research contrasting CBIS with clinical measurements during follow-up visits might be useful in HS patients.

## Supplementary Information

Supplementary Information.

## Data Availability

The data that support the findings of this study are available from the corresponding author, Dimitra Koumaki, upon reasonable request.

## Abbreviations

HS: Hidradenitis suppurativa
HRQoL: Health-related quality of life
QoL: Quality of Life
CBI: Cutaneous body image
CBIS: Cutaneous body image scale
BI: Body image
HS-PGA: Physicians’ global assessment of HS severity
MSS: Modified sartorius scale
HiSCR: Hidradenitis suppurativa clinical response
BMI: Body mass index
PMH: Past medical history
PDSAS: Patient’s disease severity assessment scale
MBSRQ: Multidimensional body-self relations questionnaire
MBSRQ-AS: Multidimensional body-self relations questionnaire-appearance scales
AE: Appearance evaluation
AO: Appearance orientation
BASS: Body areas satisfaction scale
OWP: Overweight preoccupation
SCW: Self-classified weight
DLQI: Dermatology life quality index
PHQ-9: Patient health questionnaire-9
GAD-7: General anxiety disorder-7
EQ5D 5L: EuroQol- 5D-5L
EQ VAS: Visual analogue scale
IBD: Inflammatory bowel syndrome
SD: Standard deviation
N: Number
C.I.: Confidence interval
r: Correlation coefficient
*p*value: Probability value
ISB: Internalized skin bias
