# Supplementary material for: Cutaneous body image in patients with hidradenitis suppurativa: a hospital-based cross-sectional study
Source: Sci Rep. 2023 May 16;13:7898. doi: 10.1038/s41598-023-35120-9 (PMC10188606; doi:10.1038/s41598-023-35120-9)
Supplement: Supplementary file 1 — Supplementary Information. [file 41598_2023_35120_MOESM1_ESM.doc]

**Appendix 1**

Explanation of clinical scores (instruments) used

**Cutaneous Body Image Scale (CBIS)**

Self-evaluation of cutaneous body image (CBI) was investigated using the validated CBI questionnaire, which is a seven-item scale. Please see the Supplementary Table below. The total CBIS score is derived from the mean ratings of the seven questions, where a high total count is suggestive of greater satisfaction with CBI (possible range, 0-9).

**Multidimensional Body-Self Relations Questionnaire-Appearance Scales (MBSRQ-AS)**

The 34-questionnaire version of the Multidimensional Body-Self Relations Questionnaire (MBSRQ) instrument, contains only the five appearance-related items: Appearance Evaluation (AE), Appearance Orientation (AO), Body Areas Satisfaction Scale (BASS), Overweight Preoccupation (OWP), and Self Classified Weight (SCW) items is the shortened version of the 69 questions MBSRQ attitudinal body image (BI) evaluation. It is reinforced to use this version when investigators are mainly interested in appearance-based BI. The questionnaire consists of a 5-point response format varying from 1 definitely disagree to 5 definitely agree. This study used the 34-questionnaire version of the MBSRQ-AS.

Appearance Evaluation (AE) is a 7-item measure of satisfaction with attractiveness, and positive or negative self-perceptions of physical appearance. High scores indicate positive and satisfied perceptions of physical appearance and attractiveness. Appearance orientation (AO) is a 12-item subscale measure of personal time and effort consumed on grooming and countenance. High scores express high levels of attention to appearance and extensive countenance. Overweight Preoccupation (OP) is a four-item subscale of fat concern, weight consideration, dieting, and eating restriction. High scores indicate high-fat anxiety and high levels of dieting behavior. Self-Classified Weight (SCW) consists of two items that indicate the individual’s perception of their weight— “I think I am” and the label of their weight “others say I am”. The scale ranges from 1—very underweight to 5—very overweight. High scores indicate a high subjective and internalized perception of self as obese, whilst low scores indicate a high subjective and internalized view of self as thin. The BASS is a 9-item scale that measures the degree of dissatisfaction or satisfaction with specific body areas and attributes (e.g., face, weight, muscle tone, etc.), where high scores indicate overall satisfaction with subjective body appearance, whilst low scores indicate an overall dissatisfaction with subjective body appearance. A validated Greek version was introduced by Argyrides M, et al. in 2013. The latter was used in our study.

**Health-related quality-of-life measures**

**Dermatology Life Quality Index (DLQI)**

For evaluating skin-specific HRQoL, we used the Dermatology Life Quality Index (DLQI). This is a 10-item questionnaire evaluating the impact of skin diseases on patients’ lives within the last week. Of the 10 questions, each scored 0 to 3, concern symptoms (itchy/sore/ painful/stinging), feelings (embarrassed/self-conscious), daily activities (shopping/home/garden), choice of clothes, social or leisure activities, sport, work/school, personal relationships, sexual difficulties, and the problem with taking a treatment. Every item of the questionnaire has 4 potential answers: “not at all,” “a little,” “a lot,” or “very much” which correspond to scores 0, 1, 2, and 3, respectively. The DLQI is measured by summing the score of each item, giving a maximum of 30 and a minimum of 0. The higher the score, the greater the impact of skin disorder on QoL.

**Skindex‑16**

The Skindex-16 is a health-related quality of life (HRQOL) instrument designed to measure the effects of skin disease on patients’ quality of life. Skindex-16 is a questionnaire that specifically measures the effects of skin disorders on QOL, disregarding the type of skin condition or patient's comorbidities. Derived from Skindex-29, the questionnaire consists of 16 items.

It is composed of 16 items grouped under three subscales: symptoms (questions: 1–4), emotions (questions: 5–11), and functioning (questions: 12–16). Subscale scores are converted to a linear scale of 0-100, where higher scores indicate more impaired levels of HRQoL. Comparing Skindex-16, which focuses on the degree of bother rather than frequency and is shortened based on patients’ perspectives.

**EQ-5D-5L**

We measured general General Health-Related Quality of Life, HRQoL, by using the EQ-5D-5L and EQ visual analog scale (EQ-VAS). Every item of health has 5 levels of severity (no problems, slight problems, moderate problems, severe problems, or extreme problems) and their responses are converted into a utility score ranging from 0 (death) to 1 (full health) through an established algorithm to reflect the level of a patient’s well-being. Each health state can be assigned a utility value (i.e. EQ-5D-5L index score) obtained from population studies reflecting the societal values. In absence of a national value set in Greece, the 5L value set for England developed by *Devlin et al.* was applied to generate index scores in this study ranging between -0.594 and 1. An index score of zero indicates dead and negative values represent health states worse than dead.

**Patient Health Questionnaire (PHQ-9)**

PHQ-9 is a health self-rating inventory that was developed based on the diagnostic criteria of the Diagnostic and Statistical Manual of Mental Disorders, DSM-IV. It is used to understand the time of patients suffering from nine issues comprising depression, interest reduction, and insomnia in the last two  weeks. 0 = not at all; 1 = a few days; 2 = more than half the time; 3 = almost every day. The total score of the scale is 27, where 0 to 4, 5 to 9, 10 to 14, and 15 to 27 points accordingly represent no, mild, moderate, and severe depression.

**Generalized Anxiety Disorder 7 (GAD-7)**

GAD-7 (Generalized Anxiety Disorder 7) is a 7-item anxiety questionnaire and a brief measure for evaluating generalized anxiety disorder [29].The answer options vary from 0 points (not at all) to 3 points (nearly every day). The recall period is 4 weeks; the maximum sum score is 21. A sum of ≥10 shows the presence of anxiety.

**Supplementary Τable 1:** Cutaneous Body Image (CBI) scale

| Using the following scale, please rate each of the following times with one number between 0 to 9 that best describes your response. | |
| --- | --- |
| 0 1 2 3 4 5 6 7 8 9 | |
| 0=Not all, 1-3=slightly, 4-6= moderately, 7-8= markedly, 9= very markedly | |
| Item | Rating 0 to 9 |
| 1. I like the overall appearance of my skin |  |
| 1. I like my complexion or overall colour of my skin |  |
| 1. I like the appearance of the skin of my face |  |
| 1. I like the complexion or the overall colour of the skin of my face |  |
| 1. I am very satisfied with my hair |  |
| 1. I am satisfied with the appearance of my fingernails |  |
| 1. I am satisfied with the appearance of my toenails |  |
| Cutaneous Body Image Scale (CBIS) score= average of all 7 ratings (possible range, 0 to 9) | |
| CBI dissatisfaction grading using CBIS score: score <3=severe; 3 to 6= moderate;  >6= mild to moderate | |
